# Multi-Omics Analyses of the Gut Microbiota and Metabolism in Cats with Different Body Conditions and the Effects of Fecal Microbiota Transplantation

**DOI:** 10.3390/vetsci13050436

**Published:** 2026-04-29

**Authors:** Yuchen Yao, Zixin Yang, Tianxiang Xie, Yuhe Zhang, Fuxiao Huang, Chenyuhan Meng, Yi Wu

**Affiliations:** State Key Laboratory of Animal Nutrition and Feeding, College of Animal Science and Technology, China Agricultural University, Beijing 100193, China; yaoyc231@cau.edu.cn (Y.Y.); yang_zx@cau.edu.cn (Z.Y.); 2025505610203@cau.edu.cn (T.X.); zyh0524@cau.edu.cn (Y.Z.); hfx@cau.edu.cn (F.H.); mengchenyuhan@163.com (C.M.)

**Keywords:** feline obesity, domestic cat, gut microbiota, metagenomics, serum metabolomics, fecal microbiota transplantation

## Abstract

Obesity is increasingly recognized in domestic cats and is associated with metabolic disturbances such as insulin resistance and dyslipidemia. The gut microbiota is considered an important regulator of host metabolism, yet its role in feline obesity remains unclear. In this study, a multi-omics approach was used to investigate gut microbiota composition and metabolic profiles in cats with different body conditions and to evaluate the effects of fecal microbiota transplantation (FMT) on the feline gut microbiota and overall metabolism. In Experiment 1, twenty-four cats were classified as obese, normal, or lean, and their gut microbiota and serum metabolites were analyzed. In Experiment 2, fecal microbiota from obese or lean donors were transplanted into recipient cats. Although overall microbial diversity and community structure did not differ significantly among groups, Coriobacteriaceae and *Collinsella* were enriched in obese cats, whereas Enterobacteriaceae-related taxa were more abundant in normal-weight cats. Serum metabolomics revealed alterations mainly related to amino acid and antioxidant metabolism, including O-acetylcarnitine, glutathione, and tryptophan metabolism. FMT shifted the recipient gut microbial communities toward their respective donor profiles (obese or lean) but did not significantly affect body weight or routine serum biochemical parameters during the experimental period. These findings suggest that gut microbiota remodeling may influence metabolic processes prior to detectable phenotypic changes in cats.

## Simple Summary

Domestic cats are increasingly affected by obesity, a condition that can lead to metabolic diseases and reduced well-being. Understanding the biological factors that influence body weight is important for improving feline health. One potential factor is the community of microorganisms living in the intestine, which can affect how the body processes nutrients and energy. In this study, we compared intestinal microorganisms and blood metabolic characteristics in obese, normal-weight, and lean cats, and also transferred intestinal microorganisms from donor cats with different body conditions to other cats. We found that certain intestinal bacteria were associated with different body conditions and with changes in metabolic processes in the blood. Transferring intestinal microorganisms altered the microbial communities of recipient cats, but body weight and blood indicators did not change during the study period. These results provide new insight into how intestinal microorganisms may be linked to metabolism in cats and may help guide future approaches to managing obesity in companion animals.

## 1. Introduction

In recent years, obesity in domestic cats has shown a steadily increasing prevalence, with an estimated prevalence of approximately 40% globally and as high as 63% in certain regions, such as New Zealand, largely attributable to indoor lifestyles with reduced physical activity and increased consumption of energy-dense commercial diets [1,2]. As a major nutritional disorder in companion animals, feline obesity not only compromises quality of life and longevity but is also strongly associated with insulin resistance, diabetes mellitus, hepatic lipidosis, and other metabolic complications that impose a growing burden on veterinary healthcare systems [1,3,4]. Conventional management strategies, including energy restriction, dietary modification, and increased physical activity, remain the cornerstone of treatment; however, these approaches often exhibit limited long-term efficacy and suboptimal owner adherence in clinical practice [5,6,7]. These limitations underscore the need for novel and sustainable therapeutic strategies that address the underlying biological mechanisms of obesity rather than relying solely on behavioral and nutritional interventions. Growing evidence indicates that alterations in gut microbiota composition contribute to the development of obesity and metabolic dysfunction, making microbiota-targeted interventions an emerging therapeutic avenue. FMT has therefore attracted increasing interest as a means to restore microbial homeostasis and improve metabolic outcomes, with encouraging results reported in humans and several animal models [8,9,10]. However, the reproducibility of FMT in metabolic disorders remains limited, and its efficacy in obesity has been inconsistent across studies, likely due to variations in donor selection, administration protocols, and recipient baseline characteristics [11]. Nevertheless, systematic investigations of FMT in cats remain scarce, and its efficacy, safety, and mechanistic effects in feline obesity are yet to be fully elucidated.

Obesity arises not only from an imbalance between energy intake and expenditure but is also accompanied by widespread metabolic reprogramming, characterized by dysregulation of lipid metabolism, disruption of glucose homeostasis, impaired insulin signaling, and the development of chronic low-grade inflammation [4,12]. These abnormalities interact reciprocally, promoting pathological expansion and dysfunction of adipose tissue and contributing to ectopic lipid accumulation in non-adipose organs, thereby increasing susceptibility to insulin resistance and related metabolic disorders. Accumulating evidence suggests that such disturbances extend beyond isolated biochemical alterations and reflect coordinated dysregulation of metabolic regulatory pathways that collectively impair systemic metabolic homeostasis [2,13,14]. Such complexity suggests that effective obesity interventions may require approaches capable of modulating multiple metabolic axes simultaneously rather than targeting energy balance alone.

Beyond the classical paradigm of energy imbalance, alterations in gut microbiota composition and function are increasingly recognized as important contributors to the development and progression of obesity [15]. The gut microbiota influences host energy balance and metabolic homeostasis through its roles in nutrient metabolism, short-chain fatty acid production, immune regulation, and maintenance of intestinal barrier integrity [16,17]. Through microbial metabolites and signaling molecules, it modulates lipid, glucose, and cholesterol metabolism and promotes obesity-related metabolic disturbances by shaping inflammatory responses and host metabolic signaling [18,19]. Accumulating evidence indicates that targeted modulation of gut microbiota composition can improve obesity-associated metabolic phenotypes [20,21]; however, systematic validation of these effects in cats remains limited by the small number of studies available, generally modest sample sizes, insufficient dietary standardization, and the lack of integrated multi-omics approaches capable of linking microbial alterations with host metabolic phenotypes.

Building on this background, this study integrates metagenomics and untargeted serum metabolomics to characterize gut microbial features and metabolic phenotypes in cats with different body conditions and to evaluate the effects of gut microbiota modulation on host metabolic status through FMT. This study provides multi-omics evidence supporting the association between gut microbiota and feline obesity and offers exploratory insights into microbiota-dependent metabolic and functional alterations. It also provides preliminary data for evaluating the potential applicability of fecal microbiota transplantation in modulating host microbial and metabolic profiles.

## 2. Materials and Methods

Experimental protocols of animal handling and dietary treatments were approved by the Institutional Animal Care and Use Committee of China Agricultural University (Project identification code: AW91306202-1-07).

### 2.1. Animals

Before the study, all cats underwent a comprehensive medical examination, which included assessments of blood and serum parameters, appetite, body condition, fecal score, and parasitic status.

A total of 44 sexually intact cats were enrolled in this study, including Ragdoll, Maine Coon, British Shorthair, American Shorthair, golden shaded, and domestic cats, with an equal number of males and females, aged between 1 and 7 years. All cats were healthy, with no history of immune-mediated diseases or allergies, and were free from conditions that could affect gastrointestinal function, such as liver disease, pancreatic insufficiency, metabolic disorders, parasitic infections, or renal disease. Cats that had received medications or diets related to the study objectives within the previous three months, as well as those treated with antibiotics or immunosuppressive agents or subjected to surgery, were excluded. Pregnant or lactating cats, or cats unable to feed orally or tolerate gavage, were also excluded.

During the study, all the cats were raised at the Companion Animal Science Feeding Center of China Agricultural University and received care in accordance with the guidelines of the National Research Council. The animal room was cleaned daily at fixed times to ensure proper ventilation and sanitary conditions, and disinfection was conducted according to standard biosecurity procedures. During the trial, the mental status, feed intake, and defecation of each cat were closely monitored. In addition, the cats received weekly body surface cleaning and routine deworming throughout the study period. Cats had ad libitum access to food and water, and their diet met the nutrient requirements recommended by the NRC (2006). The composition of the diet is presented in Table 1.

### 2.2. Study Design

In Experiment 1, we aimed to investigate differences in gut microbial community composition, serum untargeted metabolomic profiles, and body condition-related phenotypic characteristics among cats with different body condition scores (BCS). A total of 24 cats (4 males and 4 females per group) were divided into three groups of 8 cats each. The groups were: Obese group (OG) with BCS > 6, Normal group (NG) with 4 ≤ BCS ≤ 6, and Lean group (LG) with BCS < 4. Based on previous studies [22], the 14-day feeding period on a standard basal diet was adopted for all cats.

Experiment 2 aimed to evaluate the transmissibility and early metabolic effects of FMT derived from donors with different body conditions in recipient cats with generally normal to mildly elevated baseline body condition scores. A total of 20 adult domestic cats were enrolled as recipients and randomly assigned to two groups using a random number table (*n* = 10 per group; 5 males and 5 females each). The obese-donor FMT group (OFMT) received FMT capsules prepared from obese donor cats, whereas the lean-donor FMT group (LFMT) received FMT capsules prepared from lean donor cats. Baseline BCS of recipient cats ranged from 4 to 7, with mean values of 5.2 in OFMT and 5.3 in LFMT, indicating an overall normal to mildly overweight body condition status. All capsules were administered orally once weekly. Throughout the experimental period, all cats were maintained on the same standard basal diet, and the intervention lasted for 6 weeks (42 days).

### 2.3. Preparation of FMT Capsules

The FMT capsule preparation procedure was designed with reference to the protocols of Martini et al. [23] and Rojas et al. [24]. During Days 11–14 of Experiment 1, freshly voided fecal samples were collected from both obese and lean donor cats to prepare FMT materials. Fecal samples from obese donors were pooled in equal mass proportions to prepare the OFMT material, while samples from lean donors were pooled using the same protocol for LFMT preparation. Samples were immediately cleaned of litter, hair, and other foreign materials, lyophilized, and encapsulated into size 4 capsules, with each capsule containing approximately 80–100 mg of lyophilized fecal powder depending on packing density, which were stored at −20 °C until use.

### 2.4. Clinical Data and Sample Collection

Body weight and BCS of each cat was measured on day 0 in Experiment 1 and on days 0 and 42 in Experiment 2. Blood and fecal samples were collected at a single time point (day 14 in Experiment 1; day 42 in Experiment 2) for all animals. For each animal, after fasting for 12 h, 4 mL of blood was collected from the forelimb vein using a 5 mL vacuum collection tube and placed into a non-anticoagulant tube. After standing for 30 min to allow serum separation, the samples were then centrifuged at 3000× *g* for 10 min at 4 °C. The supernatant (serum) was stored at −20 °C. Fresh fecal samples (3–5 g per sample) were collected from each animal, transferred to cryogenic tubes, and stored at −80 °C.

### 2.5. Blood Glucose and Lipid Parameters

Blood glucose and lipid parameters, including glucose (GLU), triglyceride (TG), total cholesterol (TC), high-density lipoprotein (HDL), and low-density lipoprotein (LDL), were measured using a fully automated biochemical analyzer (Cobas 6000 c 501, Roche Diagnostics, Basel, Switzerland).

### 2.6. Fecal Metagenomic Analysis

DNA extraction from approximately 200 mg of each cat fecal sample was performed using the FastPure Stool DNA Isolation Kit (MJYH, Shanghai, China). No technical replicates were included, and each biological sample was processed independently. After DNA extraction, the integrity of the DNA was assessed using 1% agarose gel electrophoresis. The DNA was fragmented using the Covaris M220 (Gene Company Limited, Shanghai, China), and approximately 350 bp fragments were selected. Subsequently, a paired-end library was constructed using the NEXTFLEX Rapid DNA-Seq (Bioo Scientific, Austin, TX, USA).

Metagenomic sequencing was performed on the Illumina NovaSeq platform (Illumina, San Diego, CA, USA). Adapter sequences at the 3′ and 5′ ends of the reads were trimmed using fastp (version 0.23.0), and reads shorter than 50 bp or with an average base quality score below 20 were removed. Host DNA contamination was removed by aligning the filtered reads to the *Felis catus* reference genome using BWA (version 0.7.17) with default parameters. High-quality paired-end and single-end reads were retained. The optimized sequences were assembled using MEGAHIT (version 1.1.2) with the meta-sensitive preset, with contigs ≥ 300 bp selected as the final assembly result. Open reading frames (ORFs) were predicted for the contigs using Prodigal (version 2.6.3). Genes with a nucleotide length ≥ 100 bp were selected and translated into amino acid sequences. This threshold was selected to retain potentially functional short microbial coding sequences, while downstream clustering and annotation filtering were applied to reduce noise.

The predicted gene sequences were clustered using CD-HIT (version 4.6.1) with parameters of 90% identity and 90% coverage, and the longest gene from each cluster was selected as the representative sequence to construct a non-redundant gene set. High-quality reads from each sample were aligned with the non-redundant gene set using SOAPaligner (version 2.21) (95% identity), and gene abundances were normalized to relative abundances prior to downstream taxonomic and functional analyses.

The amino acid sequences of the non-redundant gene set were aligned with the NR database using Diamond (version 0.8.35) with BLASTP parameters set to an e-value of 1 × 10^−5^, retaining the top hit for taxonomic annotation. Taxonomic information corresponding to each NR hit was used to annotate species, and the abundance of each species was calculated by summing the abundances of the genes assigned to that species.

### 2.7. Serum Non-Targeted Metabolomics Analysis

A total of 100 μL of serum sample was transferred into a 1.5 mL centrifuge tube, followed by the addition of 400 μL of extraction solvent (acetonitrile:methanol = 1:1, *v*/*v*) containing four internal standards, including L-2-chlorophenylalanine (0.02 mg/mL). The mixture was vortexed for 30 s and subjected to ultrasonic extraction at low temperature (5 °C, 40 kHz) for 30 min. Subsequently, the samples were incubated at −20 °C for 30 min to precipitate proteins, followed by centrifugation at 13,000× *g* for 15 min at 4 °C. The supernatant was carefully collected and dried under a gentle stream of nitrogen gas.

The dried extracts were reconstituted with 100 μL of reconstitution solvent (acetonitrile:water = 1:1, *v*/*v*), followed by ultrasonic extraction at low temperature (5 °C, 40 kHz) for 5 min. After centrifugation at 13,000× *g* for 10 min at 4 °C, the supernatant was transferred into autosampler vials with inserts for subsequent instrumental analysis.

Serum non-targeted metabolomic analysis was performed using an ACQUITY HSS T3 column (100 mm × 2.1 mm, 1.8 μm; Waters Corporation, Milford, MA, USA) coupled with a UHPLC–Orbitrap Exploris 240 system (Thermo Fisher Scientific, Waltham, MA, USA). A 3 μL aliquot of each sample was injected. The mobile phase consisted of solvent A [water/acetonitrile (95:5, *v*/*v*) containing 0.1% formic acid] and solvent B [acetonitrile/isopropanol/water (47.5:47.5:5, *v*/*v*/*v*) containing 0.1% formic acid]. The flow rate was 0.40 mL/min, and the column temperature was maintained at 40 °C. Chromatographic grade solvents from Aladdin (Shanghai, China) were used throughout the analysis. Mass spectrometric data were acquired in both positive and negative ion modes over an *m*/*z* range of 70–1050, with spray voltages of 3500 V and −3000 V, respectively. The sheath gas and auxiliary gas were set at 50 and 13 arb, respectively, the ion source temperature was 450 °C, and stepped collision energies of 20–40–60 V were applied. Raw LC–MS data were processed using Progenesis QI software (Waters Corporation, Milford, MA, USA) for baseline filtering, peak detection, peak integration, retention time correction, and peak alignment, resulting in a data matrix consisting of retention time, mass-to-charge ratio (*m*/*z*), and peak intensity. Metabolite identification was achieved by matching MS and MS/MS spectra against public metabolite databases, including the Human Metabolome Database (HMDB) and METLIN, as well as a self-built database provided by Majorbio. The processed data matrix was subsequently uploaded to the Majorbio Cloud platform (cloud.majorbio.com, accessed date: 31 October 2025) for further analysis.

Multivariate statistical analyses were conducted using the ropls package in R software (version 1.6.2). Principal component analysis (PCA) and orthogonal partial least squares discriminant analysis (OPLS-DA) were applied to the preprocessed data matrix, and a seven-fold cross-validation procedure was used to evaluate the robustness and stability of the models.

### 2.8. Multi-Omics Correlation Analysis

Significantly different microbial taxa identified from metagenomic analysis and differential metabolites from serum non-targeted metabolomics were used for correlation analysis. Spearman correlation coefficients were calculated between microbial taxa and metabolites using the R Hmisc package (version 4.7-0), and *p*-values were adjusted for multiple testing using the Benjamini–Hochberg method. Heatmaps displaying correlations between microbial taxa and metabolites, as well as pathway-level correlations, were generated using the R pheatmap package (version 1.0.12). Multi-omics correlation networks integrating microbial taxa, metabolites, and pathways were constructed using Cytoscape (version 3.9.1) based on significant Spearman correlations (adjusted *p* < 0.05, |r| > 0.5).

### 2.9. Statistical Analysis

Microbiome sequencing data were visualized using R software. The ggplot package in R was used to generate bacterial community bar plots, and the vegan package in R was used to generate bacterial community heatmaps. Differential bacterial taxa between the two treatment groups were identified using the Wilcoxon rank-sum test with Bonferroni correction. In serum non-targeted metabolomics, significantly differential metabolites were determined based on the variable importance in projection (VIP) values obtained from the OPLS-DA model and the *p*-values from Student’s *t*-test, with metabolites having VIP > 1 and *p* < 0.05 considered as significant. These criteria were used for downstream volcano plot visualization, key metabolite screening, and KEGG pathway enrichment analyses. Given the exploratory nature of untargeted metabolomics and the relatively limited sample size of the present study, this combined threshold was used to balance statistical sensitivity and biological interpretability.

Other statistical analyses were performed using SAS 9.4 software (SAS Institute Inc., Cary, NC, USA), and visualization was performed using GraphPad Prism 9.5 (GraphPad Software, San Diego, CA, USA), with each cat considered as the experimental unit. Variables including body weight (BW), body condition score (BCS), serum glucose and lipid indices, and the relative abundance of differential taxa and pathways were first tested for normality. For normally distributed data, one-way ANOVA followed by Tukey’s honestly significant difference test was used; otherwise, non-parametric tests were applied as appropriate. Results are expressed as mean ± standard error of the mean (SEM), with *p* < 0.05 considered statistically significant and 0.05 ≤ *p* < 0.10 considered a trend.

## 3. Results

### 3.1. Phenotypic Characteristics and Metabolic Status Differences Among Cats with Different Body Conditions

**Figure 1:**
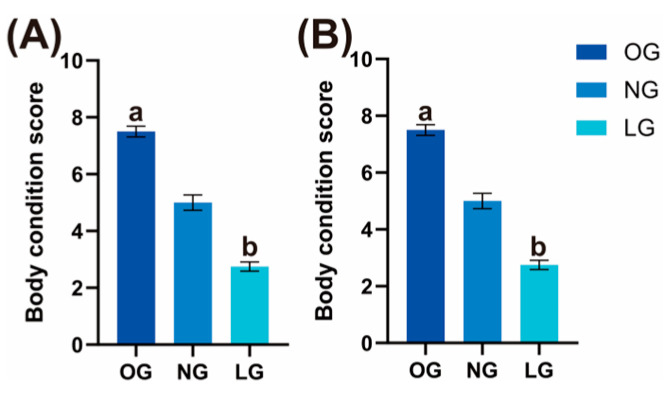
(**A**) Body weight (BW) of cats in different groups in Experiment 1; (**B**) Body condition score (BCS) of cats in different groups in Experiment 1. Data are presented as individual BCS values for each group: obese group (OG), normal group (NG), and lean group (LG). *n* = 8. a, b Values with different superscripts indicate significant differences between groups (*p* < 0.05).

The BW and BCS of cats are shown in Figure 1A,B. The BW and BCS were significantly higher in the OG than in the LG (*p* < 0.05).

### 3.2. Differences in Gut Microbiota Composition and Function Among Cats with Different Body Conditions

#### 3.2.1. Differences in Gut Microbiota Composition

**Figure 2:**
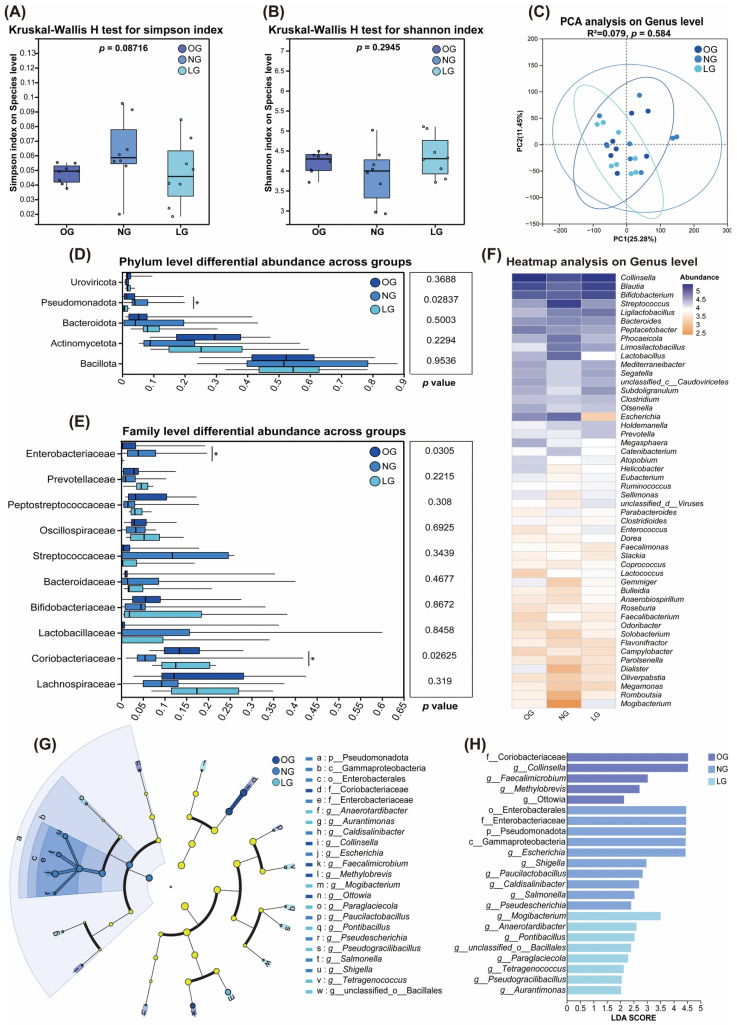
Changes in gut microbial composition of cats with different body conditions. (**A**) Alpha diversity analysis using Simpson index; (**B**) Alpha diversity analysis using Shannon index; (**C**) Principal component analysis (PCA) of gut microbial communities at the genus level; (**D**) Boxplot showing the relative abundance of the dominant bacterial taxa at the phylum level; (**E**) Boxplot showing the relative abundance of the dominant bacterial taxa at the family level; (**F**) Heatmap analysis on genus level; (**G**) Cladogram generated by LEfSe analysis; (**H**) Linear discriminant analysis (LDA) scores for taxa identified by LEfSe as differentially abundant among groups (LDA score > 2.0). OG, obese group; NG, normal group; LG, lean group. Data are presented as mean ± SEM (*n* = 8). * *p* < 0.05.

Alpha diversity analysis showed no significant differences in Simpson or Shannon indices among cats with different body conditions (*p* > 0.05), indicating overall comparable microbial richness and evenness across groups (Figure 2A,B). PCA showed no clear clustering among the NG, OG, and LG groups. Consistent with this pattern, PERMANOVA analysis based on Bray–Curtis distances revealed no significant differences in overall gut microbial community structure among groups (*p* > 0.05) (Figure 2C).

At the phylum level, gut microbial composition was relatively stable across groups and was dominated by Bacillota, Bacteroidota, Pseudomonadota, and Actinomycetota (Figure 2D). Statistical analysis showed that only Pseudomonadota differed significantly among groups (*p* < 0.05), with the highest relative abundance in NG, followed by OG and LG, whereas no significant differences were observed for other dominant phyla.

At the family level, Coriobacteriaceae and Enterobacteriaceae exhibited significant differences among groups (Figure 2E). Coriobacteriaceae showed the highest relative abundance in OG, followed by LG and NG, whereas Enterobacteriaceae was most abundant in NG, followed by OG and LG.

At the genus level, the heatmap provided the trends of the relative abundance patterns of dominant taxa across cats with different body condition states (Figure 2F). Consistent with the LEfSe results, *Collinsella* showed the highest relative abundance in the obese group, followed by the lean group and the normal group. In contrast, *Escherichia* was most abundant in the normal group, followed by the obese group and the lean group, displaying a trend opposite to body condition score.

To further identify discriminative microbial features associated with different body conditions, LEfSe analysis was performed across multiple taxonomic levels (Figure 2G,H). The results showed that OG was significantly enriched in the family Coriobacteriaceae and its genera *Collinsella* and *Faecalimicrobium*. NG was characterized by enrichment of taxa within Pseudomonadota, including the class Gammaproteobacteria, order Enterobacterales, and family Enterobacteriaceae. At the genus level, taxa assigned to the *Escherichia*/*Shigella* group and *Salmonella* were identified as discriminative features of NG. In contrast, LG was characterized by enrichment of genera such as *Mogibacterium* and *Anaerotardibacter* (LDA score > 2.0).

#### 3.2.2. Differences in Gut Microbiota Function

**Figure 3:**
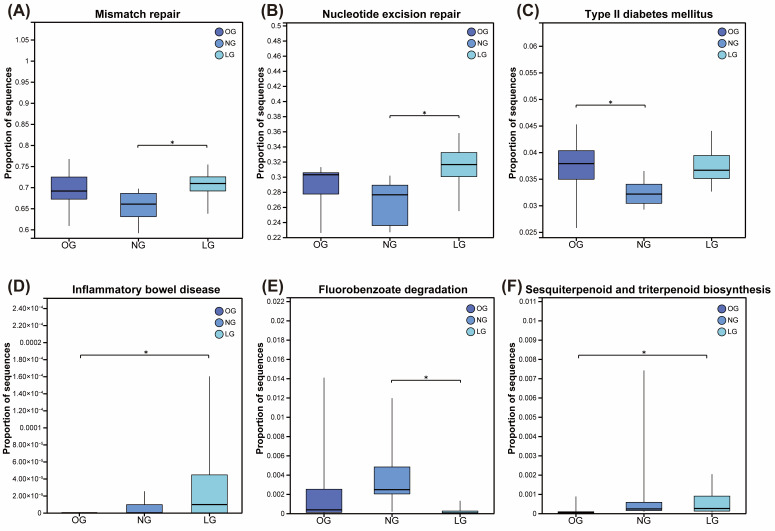
KEGG functional pathway analysis of gut microbiota in cats with different body conditions. Differentially abundant level-3 KEGG pathways among groups (*p* < 0.05). (**A**) Mismatch repair; (**B**) Nucleotide excision repair; (**C**) Type II diabetes mellitus; (**D**) Inflammatory bowel disease; (**E**) Fluorobenzoate degradation; (**F**) Sesquiterpenoid and triterpenoid biosynthesis. OG, obese group; NG, normal group; LG, lean group. Data are presented as mean ± SEM (*n* = 8). * *p* < 0.05.

Based on KEGG functional annotation, multi-group comparative analysis revealed significant differences in multiple level-3 functional categories among cats with different body conditions (*p* < 0.05). Specifically, the mismatch repair and nucleotide excision repair pathways exhibited the highest relative abundance in lean cats, followed by obese cats, and the lowest in normal-weight cats (Figure 3A,B). The type II diabetes mellitus pathway has been confirmed to be associated with insulin signaling, type II diabetes, and many other processes [25]. In the present experiment, this pathway was upregulated in both OG and LG relative to NG (Figure 3C). Likewise, the inflammatory bowel disease pathway has also been confirmed to be associated with inflammatory bowel disease (IBD), and its upregulation is further linked to mucosal immune activation, intestinal barrier regulation, and microbiota-associated inflammatory adaptation [26,27]. In the present experiment, this pathway was observed to be upregulated in both the LG and NG groups compared with the OG group (Figure 3D). In addition, fluorobenzoate degradation showed the highest abundance in normal-weight cats, followed by obese cats and lean cats (Figure 3E), whereas sesquiterpenoid and triterpenoid biosynthesis pathway was most abundant in normal-weight cats, followed by lean cats and obese cats (Figure 3F).

### 3.3. Differences in Serum Untargeted Metabolomic Profiles Among Cats with Different Body Conditions

**Figure 4:**
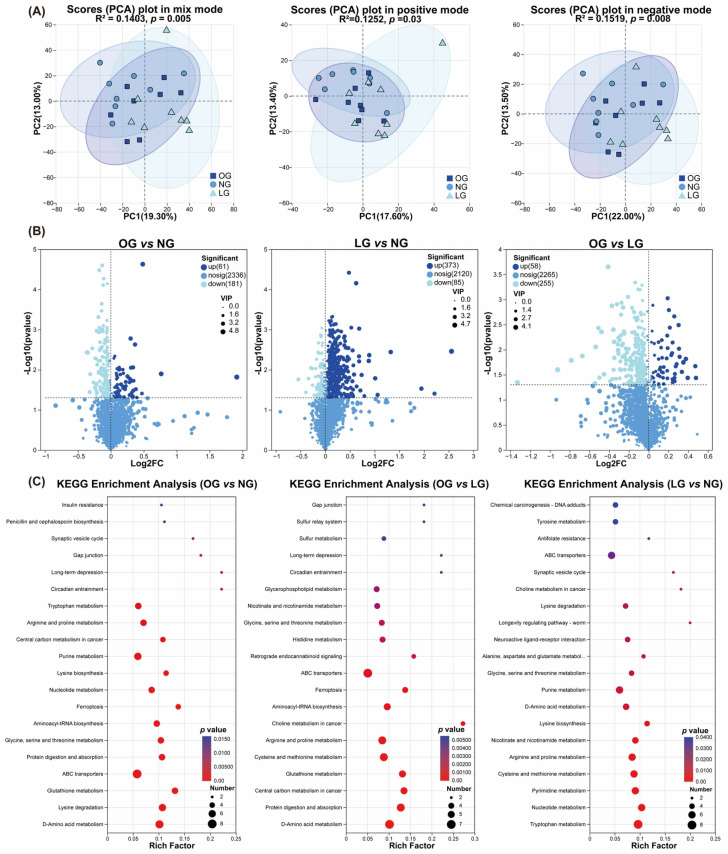
Serum metabolomic profiling and functional enrichment analysis in cats with different body conditions. (**A**) Principal component analysis (PCA) score plots of serum metabolomes in mix mode, negative ion mode, and positive ion mode; (**B**) Volcano plots displaying differential metabolites among OG vs. NG, LG vs. NG, and OG vs. LG. Metabolites with VIP > 1 and *p* < 0.05 were defined as significantly differential, with red and blue representing upregulated and downregulated metabolites, respectively, and gray indicating non-significant metabolites; (**C**) KEGG pathway enrichment analysis of differential metabolites for OG vs. LG, OG vs. NG, and LG vs. NG. OG, obese body condition group; NG, normal body condition group; LG, lean body condition group. Data are presented as mean ± SEM (*n* = 8).

PCA revealed clear separation of serum metabolomic profiles among cats with different body conditions (Figure 4A). PERMANOVA based on combined positive- and negative-ion datasets showed that body condition significantly influenced the serum metabolome (R^2^ = 0.1403, *p* = 0.005). Consistently, separate analyses of the positive- and negative-ion modes also demonstrated significant differences among groups (positive mode: R^2^ = 0.1252, *p* = 0.03; negative mode: R^2^ = 0.1519, *p* = 0.008), indicating global alterations in serum metabolic features across body condition categories.

Untargeted serum metabolomic analysis further confirmed significant differences in global metabolic profiles among cats with different body conditions (Figure 4B). Using the criteria of VIP > 1 from the predictive OPLS-DA model together with nominal *p* < 0.05, 242 differential metabolites were identified between OG and NG (61 upregulated and 181 downregulated), 458 between LG and NG (373 upregulated and 85 downregulated), and 313 between OG and LG (58 upregulated and 255 downregulated). After FDR correction, only a limited subset of metabolites remained statistically significant, indicating that the current findings should be interpreted as exploratory candidate metabolic signatures.

After applying more stringent criteria (VIP > 1.5 and *p* < 0.05) to identify key metabolites, O-acetylcarnitine (VIP = 2.1521) and stearic acid (VIP = 2.3428), both associated with energy metabolism and lipid oxidation, were significantly decreased in OG compared with NG. In addition, the L-glutathione (VIP = 2.5581) and acetylcysteine (VIP = 2.2838), which are related to the antioxidant system and sulfur-containing amino acid metabolism, were significantly upregulated. Regarding microbiota-related metabolites, tryptophan metabolism-derived indole-3-acetylglycine (VIP = 1.9914) was significantly increased in OG and were also elevated in LG compared with NG (VIP = 2.4835). In addition, the bacterial cell wall-associated metabolite diaminopimelic acid was significantly higher in both OG (VIP = 2.0423) and LG (VIP = 2.1221) relative to NG. In the comparison between OG and LG, substances related to antioxidant and sulfur-containing amino acid metabolism, such as L-glutathione (VIP = 2.3994), acetylcysteine (VIP = 1.7537) and L-cysteine (VIP = 1.7792), were further elevated in OG.

Based on the KEGG database, pathway enrichment analysis was conducted on the differential metabolites. Serum metabolic pathways in cats exhibited body condition-specific enrichment (Figure 4C). Compared with the NG group, the OG group was enriched in glutathione metabolism, arginine and proline metabolism, and tryptophan metabolism. Compared with the NG group, the LG group was enriched in cysteine and methionine metabolism, tryptophan metabolism, and nucleotide metabolism. Comparison between the OG and LG groups revealed enrichment differences in glutathione metabolism, cysteine and methionine metabolism, and glycerophospholipid metabolism.

### 3.4. Associations Between Gut Microbiota and Serum Metabolic Phenotypes

**Figure 5:**
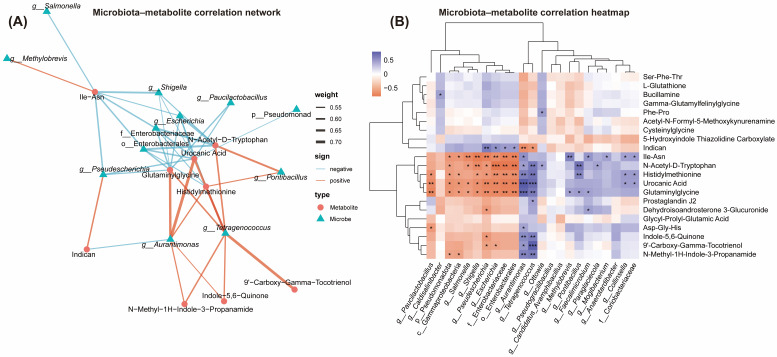
Correlation analysis between gut microbiota and high-confidence differential serum metabolites in cats with different body conditions. (**A**) The microbiota–metabolite correlation network; (**B**) The microbiota–metabolite correlation heatmap. Exhibiting Spearman’s rank correlations between gut microbial taxa and high-confidence differential metabolites (VIP > 2, *p* < 0.05). Data are presented as mean ± SEM (*n* = 8). *** *p* < 0.001; ** *p* < 0.01; * *p* < 0.05.

Exploratory correlation analysis between gut microbiota and high-confidence differential metabolites (VIP > 2, *p* < 0.05) identified across the three body condition groups were analyzed (Figure 5A,B). *Salmonella* showed significant negative correlations with isoleucine-asparagine dipeptide (Ile-Asn), N-acetyl-D-tryptophan, urocanic acid, and glutaminylglycine (ρ = −0.44 to −0.65, *p* < 0.05). *Shigella*, *Escherichia*, Enterobacteriaceae, and Enterobacterales were significantly negatively correlated with Ile-Asn, N-acetyl-D-tryptophan, histidylmethionine, urocanic acid, and glutaminylglycine (ρ = −0.45 to −0.63, *p* < 0.05). In contrast, *Tetragenococcus* was positively correlated with N-acetyl-D-tryptophan, histidylmethionine, urocanic acid, and glutaminylglycine (ρ = 0.58–0.64, *p* < 0.05).

### 3.5. Changes in Phenotypic Characteristics and Metabolic Status After FMT Intervention

**Figure 6:**
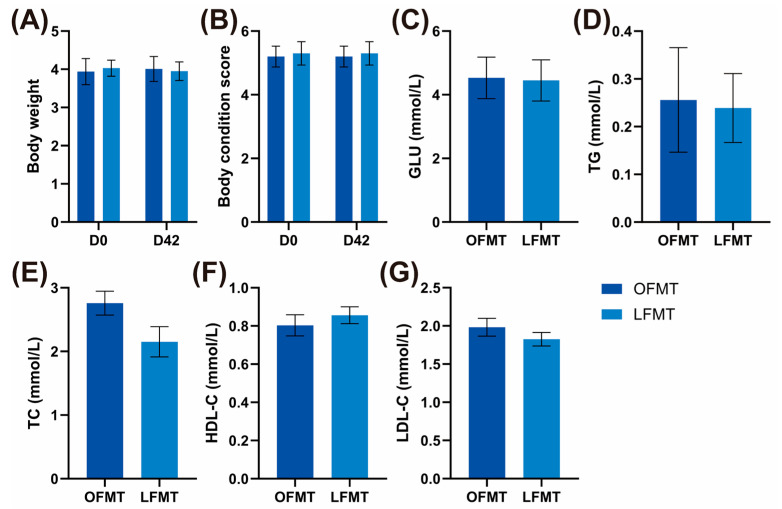
Body weight (BW), body condition score (BCS) and glucose indices of recipient cats following fecal microbiota transplantation (FMT). (**A**) Body weight (BW); (**B**) Body condition score (BCS) of cats in the obese donor FMT (OFMT) and lean donor FMT (LFMT) groups at day 0 (D0) and day 42 (D42) of the FMT trial; (**C**) Serum glucose (GLU); (**D**) Triglycerides (TG); (**E**) Total cholesterol (TC); (**F**) High-density lipoprotein cholesterol (HDL-C); (**G**) Low-density lipoprotein cholesterol (LDL-C) levels in the OFMT and LFMT groups at the end of the trial. OFMT, FMT from obese donor cats; LFMT, FMT from lean donor cats. Data are presented as mean ± SEM (*n* = 10).

The BW, BCS of cats are shown in Figure 6A,B. Baseline BW and BCS were comparable between OFMT and LFMT recipients, and both groups were within a normal to mildly overweight range prior to intervention. All of them showed no significant differences between the OFMT and LFMT groups (*p* ≥ 0.05). Additionally, in the same group, there was no significant difference in the BW and BCS at each time point (*p* ≥ 0.05).

The blood GLU, TG, TC, HDL-C, and LDL-C of cats are shown in Figure 6C–G. All of them showed no significant differences between the OFMT and LFMT groups (*p* ≥ 0.05).

### 3.6. Effects of FMT Intervention on Gut Microbiota Composition and Function

**Figure 7:**
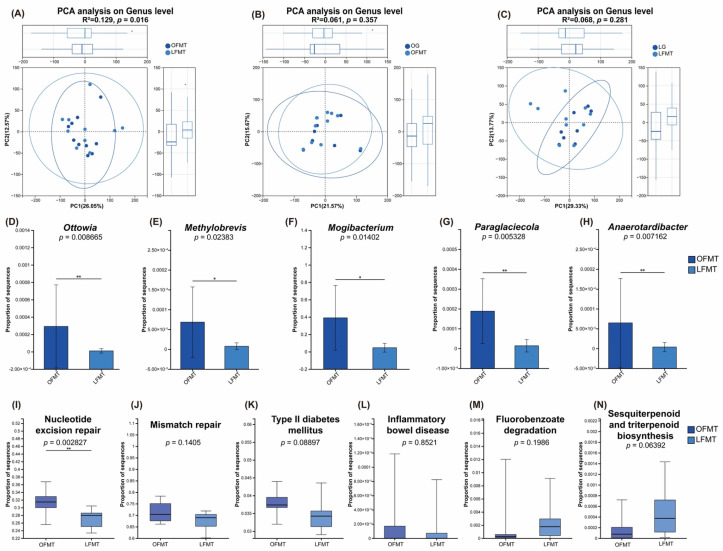
Gut microbial community structure, differential bacterial taxa and functional pathway analysis of recipient cats following FMT. Gut microbial community structure: (**A**) Principal component analysis (PCA) of gut microbial communities at the genus level comparing OFMT and LFMT groups; (**B**) PCA of gut microbial communities at the genus level comparing OFMT and OG groups; (**C**) PCA of gut microbial communities at the genus level comparing LFMT and LG groups. Differential bacterial taxa and their relative abundances in the OFMT and LFMT groups: (**D**) *Ottowia*; (**E**) *Methylobrevis*; (**F**) *Mogibacterium*; (**G**) *Paraglaciecola*; (**H**) *Anaerotardibacter*. Key KEGG functional pathways and their relative abundances in the OFMT and LFMT groups: (**I**) Nucleotide excision repair; (**J**) Mismatch repair; (**K**) Type II diabetes mellitus; (**L**) Inflammatory bowel disease; (**M**) Fluorobenzoate degradation; (**N**) Sesquiterpenoid and triterpenoid biosynthesis. OFMT, FMT from obese donor cats; LFMT, FMT from lean donor cats; OG, obese donor group; LG, lean donor group. Data are presented as mean ± SEM (n = 10 for OFMT and LFMT; *n* = 8 for OG and LG). ** *p*< 0.01; * *p* < 0.05.

PCA was performed to evaluate differences in gut microbial community structure among the groups. PERMANOVA analysis revealed a significant separation between recipients of fecal microbiota from obese donors (OFMT) and those receiving microbiota from lean donors (LFMT) (R^2^ = 0.129, *p* = 0.016) (Figure 7A). In contrast, no significant differences were observed between OFMT and the obese donor group (OG) (R^2^ = 0.061, *p* = 0.357) (Figure 7B), nor between LFMT and the lean donor group (LG) (R^2^ = 0.068, *p* = 0.281) (Figure 7C).

In Experiment 1, LEfSe analysis identified multiple bacterial taxa distinguishing the obese group (OG), normal group, and lean group (LG). Specifically, *Ottowia* and *Methylobrevis* were enriched in OG, whereas *Mogibacterium*, *Paraglaciecola*, and *Anaerotardibacter* were enriched in LG. In Experiment 2, compared with the LFMT group, the relative abundances of all five taxa were significantly increased in the OFMT group (*Ottowia*, *p* = 0.00867; *Methylobrevis*, *p* = 0.02383; *Mogibacterium*, *p* = 0.01402; *Paraglaciecola*, *p* = 0.00533; *Anaerotardibacter*, *p* = 0.00716) (Figure 7D–H).

After FMT intervention, targeted comparisons of the key KEGG functional pathways identified in experiment 1 were conducted between the OFMT and LFMT groups. Among these, only the Nucleotide excision repair pathway exhibited a statistically significant difference (*p* < 0.05), with higher abundance in the OFMT group compared to LFMT. The Mismatch repair and Type II diabetes mellitus pathways showed higher abundance in OFMT, although these differences did not reach statistical significance. In contrast, the Inflammatory bowel disease, Fluorobenzoate degradation, and Sesquiterpenoid and triterpenoid biosynthesis pathways were relatively more abundant in LFMT, but again without reaching statistical significance (Figure 7I–N).

### 3.7. Serum Untargeted Metabolomic Differences After FMT Intervention

**Figure 8:**
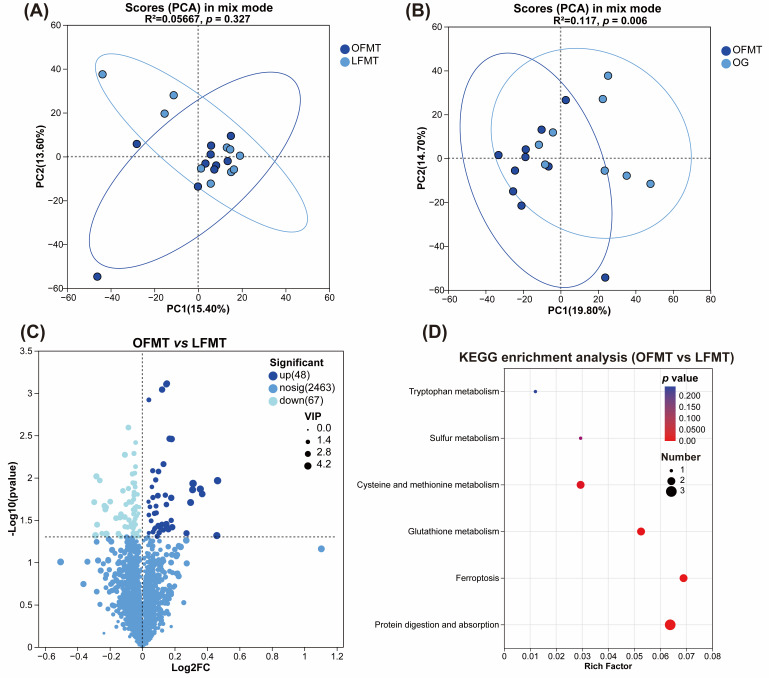
Serum metabolomic profiling and KEGG pathway enrichment analysis of recipient cats following fecal microbiota transplantation (FMT). (**A**) Principal component analysis (PCA) score plots in mix mode comparing OFMT and LFMT groups; (**B**) PCA score plots in mix mode comparing OFMT and OG groups; (**C**) Volcano plot displaying differential serum metabolites between the OFMT and LFMT groups; (**D**) KEGG pathway enrichment analysis of differential metabolites between the OFMT and LFMT groups. OFMT, FMT from obese donor cats; LFMT, FMT from lean donor cats; OG, obese donor group; LG, lean donor group. Data are presented as mean ± SEM (*n* = 10 for OFMT and LFMT; *n* = 8 for OG and LG).

PCA analysis of serum metabolomic profiles revealed no clear separation between the OFMT and LFMT groups. PERMANOVA tests confirmed the lack of statistically significant differences across the combined (R^2^ = 0.0567, *p* = 0.327) (Figure 8A), positive-ion (R^2^ = 0.0503, *p* = 0.492), and negative-ion datasets (R^2^ = 0.0626, *p* = 0.201). Comparisons between OFMT and OG showed partial separation in PCA, with PERMANOVA indicating significant differences in the combined (R^2^ = 0.1117, *p* = 0.006) (Figure 8B) and negative-ion datasets (R^2^ = 0.1254, *p* = 0.005), whereas the positive-ion dataset did not reach statistical significance (R^2^ = 0.0931, *p* = 0.057). In contrast, PCA of LFMT versus LG showed no apparent separation, and PERMANOVA tests revealed no significant differences in the combined, positive-ion, or negative-ion datasets (all *p* > 0.05). These results suggest that OFMT transplantation partially recapitulates the serum metabolic features of the obese donor group, whereas LFMT transplantation does not produce marked shifts relative to lean donors.

Non-targeted serum metabolomic analysis identified 115 differential metabolites between the OFMT and LFMT groups (VIP > 1, *p* < 0.05), of which 48 were upregulated and 67 downregulated (Figure 8C). Targeted comparison of key metabolites previously identified in Experiment 1 revealed that L-glutathione, L-cysteine and acetylcysteine were significantly increased in the OFMT group (*p* < 0.05). In contrast, O-acetylcarnitine, stearic acid, indole-3-acetylglycine and diaminopimelic acid showed no significant differences between the two groups (*p* > 0.05).

Based on the KEGG database, pathway enrichment analysis was performed for differential metabolites between the OFMT and LFMT groups (Figure 8D). Significant enrichment was observed in pathways related to protein digestion and absorption, glutathione metabolism, and cysteine and methionine metabolism (*p* < 0.05). In addition, pathways involved in one-carbon metabolism via folate, taurine and hypotaurine metabolism, and bile secretion were also significantly enriched, while D-amino acid metabolism showed significant alterations as well.

## 4. Discussion

Feline obesity is increasingly recognized as a multifactorial metabolic disorder, and microbiota-targeted interventions such as FMT have emerged as potential strategies for modulating host metabolic homeostasis. In the present study, although overall community diversity remained relatively stable across body condition groups, consistent taxonomic, metabolomic, and functional shifts were identified, supporting a body condition–associated remodeling of the feline gut microbiota.

Increasing evidence suggests that obesity-associated gut microbiota alterations are often characterized not by large-scale shifts in overall diversity, but by selective remodeling of specific taxa and functional groups [28]. Consistent with this concept, although alpha- and beta-diversity analyses in Experiment 1 did not detect significant global community differences among body condition groups, consistent directional shifts were observed at the phylum, family, and genus levels. These findings suggest that body condition–associated microbial alterations in cats may primarily occur at finer taxonomic resolution rather than through broad restructuring of the entire microbial community.

At the phylum level, members of Pseudomonadota (formerly Proteobacteria) have shown considerable variability in obesity-related studies. Several reports have linked this phylum to obesity and metabolic inflammation, and certain members, such as *Escherichia* coli, are known to promote intestinal inflammation and contribute to metabolic disturbances [28]. Studies in adolescents have suggested that abnormal body weight is closely associated with functional dysregulation of the gut microbiota, with the abundance of Pseudomonadota showing associations with metabolic abnormalities [29]. A systematic review by Crovesy et al. (2020) further indicated that human obesity is generally accompanied by an increased abundance of Pseudomonadota, although substantial interindividual variability exists and its ecological niche is strongly influenced by host, dietary, and environmental factors [30]. In our study, Pseudomonadota was the only dominant phylum exhibiting a significant change, with its relative abundance progressively decreasing from NG to OG and LG groups. This pattern implies that the ecological significance of Pseudomonadota in cats may be species-specific. Given that this phylum includes numerous Gram-negative bacteria, particularly members of the Enterobacteriaceae, whose lipopolysaccharide components can activate host immune and inflammatory signaling pathways [31], alterations in its abundance may reflect adaptive adjustments of intestinal ecological niches and dynamic regulation of the inflammatory microenvironment, rather than simply indicating metabolic stress or global microbial dysbiosis.

At the family and genus levels, we observed significant enrichment of Coriobacteriaceae and its representative genus *Collinsella* in the obese group. Previous studies have shown that these taxa participate in bile acid transformation, lipid absorption, and steroid metabolism, and their increased abundance has been associated with dyslipidemia, insulin resistance, and obesity phenotypes [31,32,33,34,35]. The enrichment of *Collinsella* observed in obese cats is therefore consistent with prior evidence and supports its potential role as a microbiota-associated marker of feline obesity.

In contrast, Enterobacteriaceae and its representative genera *Escherichia* were most abundant in the normal group and decreased in both obese and lean groups. This family has often been reported to be enriched under metabolic inflammatory conditions and is frequently regarded as comprising opportunistic or inflammation-associated bacteria, with higher abundance linked to intestinal barrier impairment and increased risk of metabolic dysfunction [36,37,38]. However, Enterobacteriaceae are facultative anaerobes capable of rapidly responding to shifts in intestinal redox gradients and utilizing alternative electron acceptors, thereby influencing intestinal redox balance. They may also contribute to maintaining efficient anaerobic conditions and limiting colonization by exogenous pathogens [38]. The higher abundance observed in normal-weight cats in our study suggests that the ecological role of Enterobacteriaceae may differ across host species or environmental contexts, rather than directly reflecting inflammatory burden or metabolic imbalance. Therefore, in cats, the ecological significance of Enterobacteriaceae may not parallel that described in human obesity models and should be interpreted cautiously in light of species-specific intestinal ecology, warranting further investigation.

Interestingly, in the present study, a microbiota–metabolite correlation network was established by integrating high-confidence differential metabolites identified in Experiment 1 with characteristic taxa detected by LEfSe analysis. The results showed that *Salmonella*, *Shigella*, *Escherichia*, Enterobacteriaceae, and Enterobacterales were significantly negatively correlated with Ile-Asn, N-acetyl-D-tryptophan, urocanic acid, and glutaminylglycine. Previous studies have shown that abnormal enrichment of Enterobacteriaceae is associated with disturbances in amino acid metabolism, as evidenced in inflammatory disease models where modulation of this bacterial family is accompanied by improvements in amino acid metabolic profiles [38,39]. In a piglet model, casein-based diets were associated with an increased fecal abundance of Enterobacteriaceae, suggesting that alterations in host diet and metabolic status may be accompanied by adaptive shifts in gut microbial composition [40]. Furthermore, in pigs subjected to a 30% lysine-restricted diet, increased abundances of *Escherichia*–*Shigella*, Aquabacterium, and Candidatus Methylomirabilis were observed, concomitant with impaired amino acid metabolism [41]. Collectively, these studies provide a relevant interpretative framework for the negative correlations observed in the present study between Enterobacteriaceae-related taxa and amino acid/dipeptide metabolites.

This interpretation is further supported by integrative microbiome–metabolome studies in obesity. Large-scale human multi-omics analyses have shown that obesity-associated microbial alterations are tightly linked to circulating amino acid and carnitine-related metabolites [42,43]. In addition, *Collinsella*, which was enriched in obese cats in the present study, has repeatedly been linked to obesity-associated metabolic dysfunction and distinct host metabolite patterns in both human and animal studies [19,44], further supporting its potential role in microbiota-driven metabolic remodeling. Moreover, as the current network is based on cross-sectional association analysis rather than direct metabolite source tracking or microbial gene–metabolite coupling, these relationships should be interpreted as biologically plausible associations rather than mechanistic evidence.

Notably, in Experiment 2, transplantation of microbiota derived from obese and lean donors resulted in a certain degree of divergence in the overall gut microbial community structure among recipient groups, with a significant difference observed between OFMT and LFMT recipients. Meanwhile, no significant differences were detected between each FMT group and its corresponding donor group. These findings suggest that gut microbial communities associated with different body conditions possess a degree of transplantability, which is consistent with previous studies in humans and animal models showing that obesity-associated microbiota can be transmitted through FMT and partially reshape metabolic phenotypes in recipients [45,46,47].

However, despite the observed microbial divergence following FMT, no significant differences were detected in BW, BCS, or serum metabolic parameters (GLU, TG, TC, HDL-C, and LDL-C) throughout the intervention period, and body condition remained stable at all time points. These findings suggest that, within the current experimental timeframe, microbial structural transferability did not immediately translate into detectable phenotypic changes.

Interestingly, an asymmetric pattern was observed in the principal component analysis of serum metabolomics in Experiment 2, suggesting that donor-dependent microbiota transfer did not fully recapitulate the overall metabolic phenotype of the obese donor group. Previous studies have shown that obesity-associated donor microbiota can induce selective microbial and metabolite alterations without completely reproducing donor-like systemic metabolic changes, particularly in recipients lacking overt obesity or metabolic dysfunction [48,49]. In the present study, recipient cats were only in a normal to mildly overweight state, which may have provided a relatively stable metabolic background and thereby limited convergence toward the serum metabolomic profile of obese donors.

Furthermore, key genera enriched in the obese and lean groups in Experiment 1, as identified by LEfSe (including *Ottowia*, *Methylobrevis*, *Mogibacterium*, *Paraglaciecola*, and *Anaerotardibacter*), were also significantly elevated in the corresponding donor-derived recipient groups in Experiment 2, indicating successful engraftment following transplantation. Given that functional characterization of several of these genera in the context of animal gut metabolism remains limited, the present findings primarily support their stable association with body condition status and partial transmissibility, while their specific mechanistic contributions warrant further validation.

Serum metabolomic profiling further demonstrated clear separation among cats with different body condition states in Experiment 1. Differential metabolites were mainly enriched in pathways related to lipid oxidation, sulfur-containing amino acid metabolism, and tryptophan metabolism, suggesting systemic remodeling of energy metabolism and redox status. As only a limited number of metabolites remained significant after FDR correction, the following interpretations should be considered hypothesis-generating.

O-acetylcarnitine was significantly lower in the obese group compared with the other groups. Previous studies have reported that o-acetylcarnitine levels may increase under obese conditions, indicating alterations in lipid and glucose metabolism [50]. As an acylcarnitine intermediate in mitochondrial fatty acid β-oxidation, its circulating level is closely linked to mitochondrial function and insulin resistance [51,52,53,54]. This contrasts with the decrease observed in the present study. The reduced O-acetylcarnitine level in obese cats may therefore reflect species-specific metabolic characteristics or alternative regulatory mechanisms requiring further investigation.

Meanwhile, L-glutathione, acetylcysteine, and L-cysteine were significantly elevated in the obese group, and KEGG enrichment analysis revealed significant upregulation of glutathione metabolism and cysteine and methionine metabolism pathways. Glutathione is a key antioxidant molecule responsible for maintaining cellular redox homeostasis [55]. Previous studies have shown that obesity is associated with reduced activity of major enzymatic antioxidant systems, including catalase (CAT), superoxide dismutase (SOD), glutathione peroxidase (GPx), and glutathione reductase (GRd) [32], resulting in increased oxidative stress [56]. Under such conditions, enhanced glutathione synthesis may represent a compensatory response aimed at restoring redox balance. Moreover, γ-glutamylcysteine—synthesized from L-glutamate and L-cysteine—constitutes the rate-limiting step in glutathione biosynthesis, and its upregulation has been associated with oxidative stress conditions [57]. The coordinated elevation of glutathione-related metabolites and pathways in the obese group is therefore consistent with compensatory antioxidant mechanisms reported in other animal models of metabolic stress.

Regarding tryptophan metabolism, indole-3-acetylglycine was significantly increased in both obese and lean groups relative to the normal group. Because gut microbiota-mediated tryptophan metabolism generates multiple indole derivatives closely associated with obesity-related metabolic dysfunction [58,59], this finding may indicate altered microbiota–host co-metabolism across divergent body condition states.

In addition, diaminopimelic acid (DAP) was elevated in both obese and lean groups relative to the normal group. As a structural component of bacterial DAP-type peptidoglycan that is not synthesized by the host [60,61], DAP may serve as a microbial-derived metabolic signature. Its elevation in cats with abnormal body condition therefore supports the possibility of altered microbiota-associated metabolic homeostasis. However, given the exploratory design of the untargeted metabolomics analysis and the VIP plus nominal *p*-value–based screening strategy, these metabolite alterations should be interpreted as candidate signatures requiring targeted validation in larger cohorts.

In Experiment 2, no significant separation was observed between the overall metabolomic profiles of the OFMT and LFMT groups. However, a significant difference emerged between the OFMT group and the original obese donor group, indicating that OFMT did not fully recapitulate the global metabolic phenotype of obese donors. Nevertheless, OFMT exhibited directional changes consistent with obese donors in metabolites associated with glutathione and sulfur-containing amino acid metabolism. Importantly, although no significant differences were detected between OFMT and LFMT in BW, BCS, or serum lipid and glucose parameters, the OFMT group showed significant increases in L-glutathione, L-cysteine, and acetylcysteine, with enriched pathways again concentrated in glutathione metabolism and cysteine and methionine metabolism, mirroring the pattern observed in obese cats in Experiment 1. These findings suggest that microbiota derived from obese donors may modulate host redox metabolic pathways prior to the emergence of overt body condition phenotypes, without inducing short-term alterations in body weight or circulating lipid profiles. The coordinated upregulation of antioxidant and sulfur amino acid-related metabolites in the OFMT group provides further evidence supporting a microbiota-mediated remodeling of host redox metabolism.

To further explore whether these metabolic alterations were accompanied by functional shifts in the gut microbiota, metagenomic functional annotation was performed. In the present study, significant differences were identified in multiple KEGG level 3 functional pathways of the gut microbiota among cats with different body condition states. These pathways were primarily enriched in DNA damage repair (Mismatch repair and Nucleotide excision repair), metabolism-related disease pathways (Type II diabetes mellitus), and inflammation- and environmental stress-associated pathways (Inflammatory bowel disease, Fluorobenzoate degradation, and Sesquiterpenoid and triterpenoid biosynthesis). Notably, part of these functional alterations exhibited a transferable trend following FMT intervention in Experiment 2, suggesting that microbial functional architecture may contribute to the establishment of host metabolic states associated with body condition.

Previous studies have reported that increased abundance of microbial DNA damage repair pathways is often interpreted as an adaptive response of the gut microbiota to oxidative stress or inflammation-related environmental pressure and has been closely linked to obesity and metabolic inflammation [62,63,64,65]. In the present study, both Mismatch repair and Nucleotide excision repair were elevated in lean and obese cats compared with normal controls. In Experiment 2, Nucleotide excision repair was significantly higher in the OFMT group than in the LFMT group, indicating that obesity-associated microbiota may exist in a heightened genomic stress repair state. This observation is consistent with prior reports describing an oxidative and inflammatory tension-enriched microenvironment in obese gut ecosystems.

In addition, the Type II diabetes mellitus pathway was enriched in both obese and lean groups relative to the normal group in Experiment 1 and exhibited a higher abundance in the OFMT group in Experiment 2. Although this pathway has been confirmed to be associated with Type II diabetes, its enrichment did not imply that the cats had the disease; instead, it merely indicates a statistical association at the metabolite pathway level. Such enrichment suggests a potential capacity of the gut microbiota to indirectly influence host metabolic homeostasis through metabolite production or inflammation-mediated pathways. Alterations in this pathway have been repeatedly documented in obese individuals and in high-fat diet-induced animal models [66,67]. Considering that OFMT did not induce significant changes in GLU or lipid parameters in Experiment 2, these findings imply that microbial functional shifts may precede measurable alterations in host clinical metabolic indices. Collectively, the present results support a stable association between gut microbial functional profiles and host glucose–lipid metabolic regulation.

The Inflammatory bowel disease pathway was most enriched in lean cats in Experiment 1 and showed a higher abundance trend in the LFMT group in Experiment 2. Similarly, the upregulation of the Inflammatory bowel disease pathway—which has also been confirmed to be associated with IBD—is further linked to mucosal immune activation, intestinal barrier regulation, and microbiota-associated inflammatory adaptation [26,27]. The present findings suggest that, under lean conditions, the gut microbial functional configuration may be more strongly characterized by immune and barrier-regulatory modules, potentially reflecting distinct ecological pressures within the intestinal niche across different body condition states. By the same token, it should be noted that enrichment of this pathway reflects a statistical association at the metabolite pathway level and does not imply that the cats had inflammatory bowel disease.

Despite these findings, several limitations of the present study should be acknowledged. First, the current study was designed as a controlled exploratory animal experiment rather than a large-scale population study. Although the sample size used here is generally consistent with similar companion animal intervention studies, the relatively limited number of animals may reduce the statistical power of multi-group comparisons and multivariate analyses, including PERMANOVA and OPLS-DA, thereby increasing the possibility of both false-negative and false-positive findings. Consequently, community-level clustering patterns, differential metabolite screening results, and microbiota–metabolite correlation network structures should be interpreted with caution, and their generalizability remains limited. In addition, although FMT-induced alterations were observed at both the microbiome and metabolome levels, no overt host phenotypic changes were detected, which may be attributable to the relatively limited 6-week intervention period. Future longer-term longitudinal studies are still required to determine whether these microbial and metabolic shifts can ultimately translate into measurable phenotypic outcomes.

In summary, the present study demonstrates that body condition-associated alterations in the feline gut microbiota are characterized by coordinated changes at the compositional, metabolic, and functional levels. Structurally, distinct microbial taxa were enriched across body condition groups and partially transferable through FMT. Metabolically, alterations in amino acid-related metabolites and redox-associated molecules suggest systemic metabolic remodeling linked to microbial variation. Functionally, the enrichment of DNA damage repair pathways, metabolism-related modules, and immune–environmental stress pathways indicates that microbial communities under different body conditions may operate under distinct ecological and metabolic pressures. Importantly, several structural and functional features were consistently reproduced following FMT, supporting the potential transmissibility and stability of body condition–associated microbial signatures. Although overt phenotypic shifts were not observed within the intervention period, these findings collectively suggest that gut microbiota remodeling may precede or contribute to metabolic state transitions in cats, providing a foundation for future mechanistic and longitudinal investigations.

## 5. Conclusions

In this study, metagenomic sequencing and serum metabolomics were integrated to investigate gut microbiota and metabolic characteristics in cats with different body conditions and to evaluate the effects of fecal microbiota transplantation. Obese cats showed enrichment of Coriobacteriaceae and *Collinsella*, whereas *Enterobacteriaceae*-related taxa were more abundant in normal-weight cats, accompanied by alterations in amino acid and antioxidant metabolism. Transplantation of fecal microbiota from donors with different body conditions altered the gut microbial community structure of recipient cats, although no significant changes in body weight or serum metabolic parameters were observed during the experimental period. These findings suggest that gut microbiota may contribute to metabolic regulation in cats and highlight the need for further studies to clarify its role in feline obesity and metabolic health.

## Figures and Tables

**Table 1:** Dietary composition and nutritional level of basal diet.

Diet Composition	%	Nutrient Content	%
Chicken meal	54.50	Moisture	7.12
Chicken fat	8.00	Crude protein	41.65
Fish oil	2.00	Crude fat	20.28
Tapioca	3.00	Crude fiber	1.82
Potato starch	19.00	Ash	7.87
Rice	4.00		
Chicken liver powder	5.00		
Alfalfa meal	3.00		
Choline chloride	0.30		
Salt	0.50		
Taurine	0.20		
Mineral complexes and vitamins ^1^	0.50		

^1^ Mineral complexes and vitamins provided the following per kilogram of feed: vitamin A (14,500 IU), vitamin D_3_ (1000 IU), vitamin E (156 IU), and vitamin B_1_ (32.0 mg), vitamin B_2_ (30.0 mg), vitamin B_3_ (120 mg), vitamin B_5_ (88.0 mg), vitamin B_6_ (13.0 mg), vitamin B_12_ (0.20 mg); Fe (FeSO_4_) 100 mg, Cu (CuSO_4_) 7.00 mg, Co (CoSO_4_) 1.00 mg, I (CaI_2_) 20.0 mg, Mn (MnSO_4_) 20.0 mg, Zn (ZnSO_4_) 68.0 mg, and Se (Na_2_SeO_3_) 0.50 mg.

## Data Availability

The original contributions presented in this study are included in the article. Further inquiries can be directed to the corresponding author.
